# Role of Selected Genetic Polymorphisms in the Development of Rheumatoid Arthritis in a British White Population

**DOI:** 10.3390/genes15081009

**Published:** 2024-08-01

**Authors:** Sarabjit Mastana, Ella Knight, Abigail Hampson, Liz Akam, David John Hunter, Anant Ghelani, Ash Samanta, Puneetpal Singh

**Affiliations:** 1 School of Sport, Exercise and Health Sciences, Loughborough University, Loughborough LE11 3TU, UK; ellaknight@rocketmail.com (E.K.); abigailhampson1@hotmail.com (A.H.); e.c.akam@lboro.ac.uk (L.A.); d.j.hunter@lboro.ac.uk (D.J.H.); anantghelani@hotmail.com (A.G.); 2 Rheumatology, University Hospitals of Leicester NHS Trust, Leicester LE1 5WW, UK; a.samanta@talk21.com; 3 Department of Human Genetics, Punjabi University, Patiala 147002, India; puneetpalsingh@pbi.ac.in

**Keywords:** Polygenic Risk Score (PRS), *TNFα*, *HLADRB1*, *PTPN22*, *TRAF1*, *IL4590*

## Abstract

Background: Rheumatoid arthritis (RA) is a complex autoimmune disease that negatively affects synovial joints, leading to the deterioration of movement and mobility of patients. This chronic disease is considered to have a strong genetic inheritance, with genome-wide association studies (GWAS) highlighting many genetic loci associated with the disease. Moreover, numerous confounding and non-genetic factors also contribute to the risk of the disease. Aims: This study investigates the association of selected genetic polymorphisms with rheumatoid arthritis risk and develops a polygenic risk score (PRS) based on selected genes. Methods: A case-control study recruited fully consenting participants from the East Midlands region of the UK. DNA samples were genotyped for a range of polymorphisms and genetic associations were calculated under several inheritance models. PRS was calculated at crude (unweighted) and weighted levels, and its associations with clinical parameters were determined. Results: There were significant associations with the risk of RA at six genetic markers and their associated risk alleles (TNRF2*G, TRAF1*A, PTPN22*T, HLA-DRB1*G, TNFα*A, and IL4-590*T). The TTG haplotype at the VDR locus increased the risk of RA with an OR of 3.05 (CI 1.33–6.98, *p* = 0.009). The GA haplotype of *HLADRB1*-*TNF*α-308 was a significant contributor to the risk of RA in this population (OR = 2.77, CI 1.23–6.28, *p* = 0.01), although linkage disequilibrium was low. The polygenic risk score was significantly higher in cases over controls in both unweighted (mean difference = 1.48, t_285_ = 5.387, *p* < 0.001) and weighted (mean difference = 2.75, t_285_ = 6.437, *p* < 0.001) results. Conclusion: Several genetic loci contribute to the increased risk of RA in the British White sample. The PRS is significantly higher in those with RA and can be used for clinical applications and personalised prevention of disease.

## 1. Introduction

**Figure 1:**
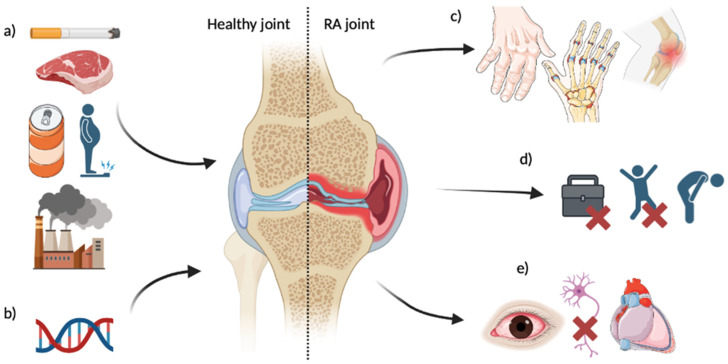
Overview of identified risk factors for RA development and disease impacts. (**a**) Lifestyle factors; (**b**) Genetic factors—see Table 1; (**c**) Affected joint impacts—may include deformity, swelling, pain, limited ROM [2]; (**d**) Quality of life impacts—may include cessation of work [4], lower functional ability, fatigue [1]; (**e**) Systemic impacts—may include eye inflammation [8], nerve compression [9], pericarditis [10]. RA risk factors and impacts are not limited to the above.

RA is a chronic autoimmune disease which currently has no cure. RA impacts the joints through irreversible progressive joint destruction. As the immune system attacks the cells lining joints, symptoms include swelling (particularly in peripheral joints), pain, stiffness, deformity, and fatigue [1,2]. RA costs the National Health Service of the UK GBP ~560 million per year [3], with 1.5 men and 3.6 women per 10,000 individuals developing the disease each year in England. The burden upon quality of life is significant, with 1/3 of patients ceasing work within 2 years of onset [4]. The disease is multifactorial, with several lifestyle factors displaying associations with RA risk (Figure 1), as well as approximately 60% of susceptibility being attributed to genetic variance [5,6,7]. 

The main manifestations of RA are synovitis and pannus formation, which lead to damage of the bone and cartilage. As soon as 4 months post-onset, bone oedema and erosive activity can be identified [11], with active synovitis in peripheral joints before presentation of clinical symptoms [12]. Layers of type A and fibroblast-like synoviocytes (FLS) in the synovial lining lead to hyperplasia as the disease progresses [1], with increased B cell, T cell, macrophage, and plasma cell numbers in the sub-lining also. This presents as joint swelling and pain in patients.

FLS located in the intimal synovium have a role in regulating joint structure; however, in excess, they act as inflammation mediators. In RA patients, FLS reduce apoptosis, contributing to pannus formation [13]. Pannus is an invasive synovial tissue which is initially composed of mononuclear cells and fibroblasts [14,15], being replaced as the disease progresses by fibrous pannus derived from collagen and pannus cells which sit above the cartilage [16]. Pannus is linked to the destruction of cartilage and bone [17], resulting in deformity and limiting range of motion [2]. While treatments slowing the progression of RA are available, including disease-modifying anti-rheumatic drugs (DMARDS) as a first-line treatment and biologics such as TNF-α inhibitors if required [4], no cure currently exists. When uncontrolled, systemic effects of RA may include inflammation of the eyes [8], nodules in the organs [18], pericarditis [10], ‘Felty’s syndrome’ [19], and losses of sensory and motor function due to peripheral nerve compression by pannus [9].

In addition to age and sex, there are several modifiable lifestyle factors like alcohol consumption, smoking, and diet which may modulate RA risk in different populations due to variable cultural and nutritional practices.

Genetics account for over half of RA risk [5,6], with a greater disease concordance observed in monozygotic twins (12–15%) than dizygotic twins (4%) [20,21], reinforcing the genetic contribution. *HLA-DBR1* alleles are significant contributors to RA risk—within this region, variants of the ‘shared epitope’ sequence of alleles are strongly associated with both RA incidence and severity due to an autoimmune response which results from specific allele combinations [22]. Polymorphisms within the *PTPN22* gene have also been strongly associated with RA [23,24]—this gene and *HLA-DRB1* have been proposed to account for 50% of the total genetic risk for RA [25]. Several gene variants appearing to contribute to RA risk have been identified, often through genome-wide association studies (GWAS); however, many of these present mixed or contrasting associations with the disease in diverse populations. Identifying genetic differences is of increasing importance as genetic screening becomes more widespread [26], and can offer lifestyle modifications for high-risk individuals as well as personalised medicine in the future.

This study focused on the analysis of 11 single-nucleotide polymorphisms (SNPs) of nine genes to develop a polygenic risk score for a British White population from the East Midlands of the UK. Details of the analysed loci are provided in Table 1.

**Table 1:** Selected genes, SNPs, and their biological roles and associations with RA.

Gene/SNP	SNP ID and Polymorphic Change	Biological/Aetiological Features	Association with Rheumatoid Arthritis	Source
Angiotensin-converting enzyme (*ACE*) gene	rs4646994 insertion/deletion (INDEL) polymorphism	*ACE* gene catalyses the conversion of angiotensin I to angiotensin II. Ang II is a powerful proinflammatory mediator seen in autoimmune diseases such as RA. AngII can also induce cell growth and proliferation, contributing to the pannus seen in RA joints.	An I/D deletion at intron 16 has been proven to increase serum ACE activity in arthritic joints, with the D/D genotype possessing the highest level of ACE activity. This therefore suggests that those with the D/D genotype are more susceptible to the disease, and at a higher severity.	(Dwivedi et al., 2018) [27]
Vitamin D receptor (*VDR*) Bsm1	rs1544410 b/B (now C/T) polymorphism	The *VDR* gene is composed of several exons and introns, spanning 75 kb. Several SNPs have been located, including BsmI, FokI, and TaqI, named according to the restriction enzyme required for their detection. Vitamin D plays a role in the activation of macrophages, enhanced production of Th1 cytokines, and decreased production of Th2 cytokines. Th1 cytokines are crucial in auto-immune responses and disorders.	The VDR BsmI polymorphism lacks research with regards to RA, and many relationships between Bsm1 and RA are currently hypothetical. However, a C/T polymorphism has been associated with increased bone loss and decreased bone mineral density, with the T/T genotype proving increased levels of these effects.	(Rass et al., 2006) [28]
VDR Fok1	rs10735810 f/F (now T/C) polymorphism		T/C polymorphism has been reported to be more common in RA patients than controls. A nucleotide change seen in this SNP has been reported to change tryptophan with arginine in *VDR*s, altering vitamin D binding sites.	(Karray et al., 2012) [29]
VDR Taq1	rs731236 A/T polymorphism		A/T polymorphism has been associated with RA in surrounding studies. However, statistically significant associations are debated and varied between ethnicities. Therefore, it can be suggested that Taq1 may contribute to RA, and when coinherited with other *VDR* polymorphisms, it may contribute to susceptibility to the disease.	(Maalej et al., 2005) [30]
Tumour necrosis factor receptor 2 (*TNFR2*)	rs1061622 M/R (now T/G) polymorphism	*TNF-α* plays an important role in pro-inflammatory responses and is mediated through two receptors, TNRFI and TNRFII. They are both expressed in many joint components in patients with RA.	It has been widely reported that a T/G polymorphism has significant association with RA risk. This polymorphism leads to a change in the amino acid within the *TNRF11*. This contributes to a stronger TNF-α signal and therefore an increased pro-inflammatory response.	(Song, Bae, and Lee, 2014) [31]
Interleukin-4 (*IL4*)-590	rs2243250 C/T polymorphism	IL4 is a cytokine produced by CD4+ lymphocytes, and it improves the anti-inflammatory response and protects against cartilage and bone destruction in type II collagen-induced arthritis.	A T/C promoter polymorphism (−590) has been associated with increased susceptibility to RA. SNP has shown significant association with the disease in several studies and meta-analyses.	(Park et al., 2017) [32]
Single transducer and activator of transcription 4 (*STAT4*)	rs7574865 G/T polymorphism	STAT4 mediates the signalling of several cytokines such as interleukins and interferons. It is said to have a crucial role in the differentiation and proliferation of T helper cells.	A G/T polymorphism has been reported to have a significant association with RA in several populations. The T allele has been associated with anti-CCP antibodies and RF positivity.	(Gao et al., 2020) [33]
TNF receptor-associated factor 1 (*TRAF1*)	rs10818488 G/A polymorphism	TRAF1 encodes proteins that mediate TNF-a single transduction, which is associated with T-cell proliferation and activation. C5 is a key member of the complement pathway in the immune response, which has been highlighted in association with RA.	The G/A polymorphism links to an intergenic region which influences the structure, function, and expression of *TRAF1* or *C5.*	(Zhang and Xiao, 2017) [34]
Protein tyrosine phosphatase non-receptor type 22 (PTPN22)	rs2476601 C/T polymorphism	*PTPN22* codes for a regulator enzyme that exhibits activity for T and B cells	The C/T polymorphism leads to a change from tryptohan to arginine, increasing T-cell activation.	(Hinks, Worthington, and Thomson, 2006) [35]
Human leukocyte antigen DR isotope 1 (HLA-DRB1)	rs660895 A/G polymorphism	The *HLA-DRB1* gene is part of the human leukocyte antigen (HLA) complex, which has been extensively associated with RA and other autoimmune diseases. HLAs play a vital role in immunity and disease susceptibility.	DRB1 alleles play role in shaping T-cell receptors and/or auto-immune responses. Studies highlight that an A/G polymorphism within this locus contributes to an increased risk of RA. Some studies also suggest protective associations.	(Das, Baruah, Saikia, and Bose, 2017) [36]
Tumour necrosis factor α (TNF-α)	rs1800629 G/A polymorphism	TNF-α has been suggested to be a potent signalling molecule, inducing other pro-inflammatory molecules. It is produced by cells present in rheumatoid arthritis joints and regulates the production of IL-1. TNF receptors have a similar distribution in RA joints, suggesting that TNF can stimulate excessive upregulation of pro-inflammatory responses.	A G/A polymorphism has been associated with an increased risk of RA, with AA and AG genotypes showing higher erosion scores. However, the association of this polymorphism has been debated.	(Korczowska, 2014) [37]

## 2. Materials and Methods

This study assessed the associations of 11 genetic loci and associated SNPs/INDELs with the risk of developing rheumatoid arthritis using a sample of 150 controls and 137 patients of European descent. The details of recruitment and some molecular genetic analyses have also been previously reported [38]. The data collection and analysis were approved by Loughborough University and hospital ethics committees [38]. All participants provided written consent, and RA patients were diagnosed by consultant rheumatologists using ARA criteria. The RA consultants only recruited RA cases who fulfilled the 1987 American Rheumatism Association criteria, had established long-term RA, and were rheumatoid-factor positive. Patients fulfilled at least four of seven ARA criteria. Inclusion criteria included patients (a) of European origin settled in the East Midlands area of the UK, (b) who were established and clinically confirmed following ARA and RF positive status, (c) and who did not have any other musculoskeletal disorders. The exclusion criteria included (a) those affected with other diseases and not classified as having RA by clinicians. All controls were free of RA symptoms. The duration of disease ranged from 2 years to 50 years in this sample. Most RA patients had been using Aspirin, steroids, DMARDs, or a combination of these drugs for a long time (2–15 years). The length of the medication use and the duration of the disease matched well. It was not possible to sufficiently separate all the treatment groups due to undergoing multiple drug therapy. The current study analysed six new polymorphisms (*STAT4*, *IL4-590*, *TRAF1-C5*, *PTPN22*, *HLADBR1*, and *TNF⍺*-308) and developed a polygenic risk score based on 11 loci.

Statistical Analysis: This study uses a case-control design. Unpaired t-tests were used to identify significant demographic or lifestyle covariates, and Hardy–Weinberg equilibrium (HWE) was tested for before further analysis. SNPstats [39] software was used to test for disease–SNP associations, producing crude odds ratios (ORs) and adjusted ORs. Bonferroni correction (modified *p* value = 0.0045 instead of 0.05) was applied where appropriate due to multiple comparisons [40]. Crude and adjusted polygenic risk scores were also calculated to indicate the combined effect of risk alleles.

The polygenic risk scores were calculated in Microsoft Excel version 2406, where:

- Homozygous without the presence of the risk allele = 0
- Heterozygous with the presence of one risk allele = 1
- Homozygous with the presence of two risk alleles = 2

Each participant therefore had an individual total risk for the ensemble of the selected polymorphisms (unweighted polygenic risk score), which was then multiplied by the relevant allelic odds ratios to work out the weighted polygenic risk score.

## 3. Results

Participant characteristics and lifestyle variables for patients and controls are presented in Table 2. Significant differences were observed for all variables; therefore, these covariates are adjusted in genetic association analyses.

Allele frequencies, genotype frequencies, and Hardy–Weinberg equilibrium *p*-values were computed and are listed in Table 3. Results with an HWE *p*-value of 0.05 or lower indicate a departure from the Hardy-Weinberg equilibrium, which was observed for both patients and controls for the STAT 4 locus and in controls for the ACE locus in this sample; therefore, association analyses at these loci should be interpreted with caution.

Table 4 lists the genetic association statistics for different loci using multiple models. Both crude and adjusted odds ratios (adjusted sex, age, diet, smoking status, and alcohol consumption) are included in this table. At the ACE locus, the codominant model highlights that the ACE I/D genotype is protective (OR 0.43, CI 0.23–0.80, *p* = 0.006). However, there is no significant association with any other model of inheritance. At VDR and STAT4 loci, none of the SNPs were associated statistically with RA, though some of the odds ratios were above 1.

Significant genetic associations were observed for TRAF-C5, IL4-590, TNFR2, HLADBR1, and PTPN22 in both crude and adjusted models (Table 4). At the TNF-⍺ 308 locus the crude ORs were statistically significant but lost significance once adjusted.

The haplotypes were computed for the VDR locus (VDRBsmI, VDRFokI, and VDRTaqI). Eight haplotypes were identified with frequencies above 1%. The linkage disequilibrium at this locus was low (D’ VDR FokI—VDRTaqI = 0.146), and none of the associated *p* values were significant. The ATA haplotype was most common, with a frequency of 0.185. The TTG haplotype was the only significantly susceptible haplotype, with an OR of 3.05 (CI 1.33–6.98, *p* = 0.009). Similarly, haplotype analysis of the HLADRB1 and TNF loci showed a very low level of linkage disequilibrium (D’ = 0.016, *p* = 0.75). The GA haplotype (HLADRB1-TNFa-308) was a significant contributor to the risk of RA in this population (OR = 2.77, CI 1.23–6.28, *p* = 0.01)

### Polygenic Risk Scores

**Figure 2:**
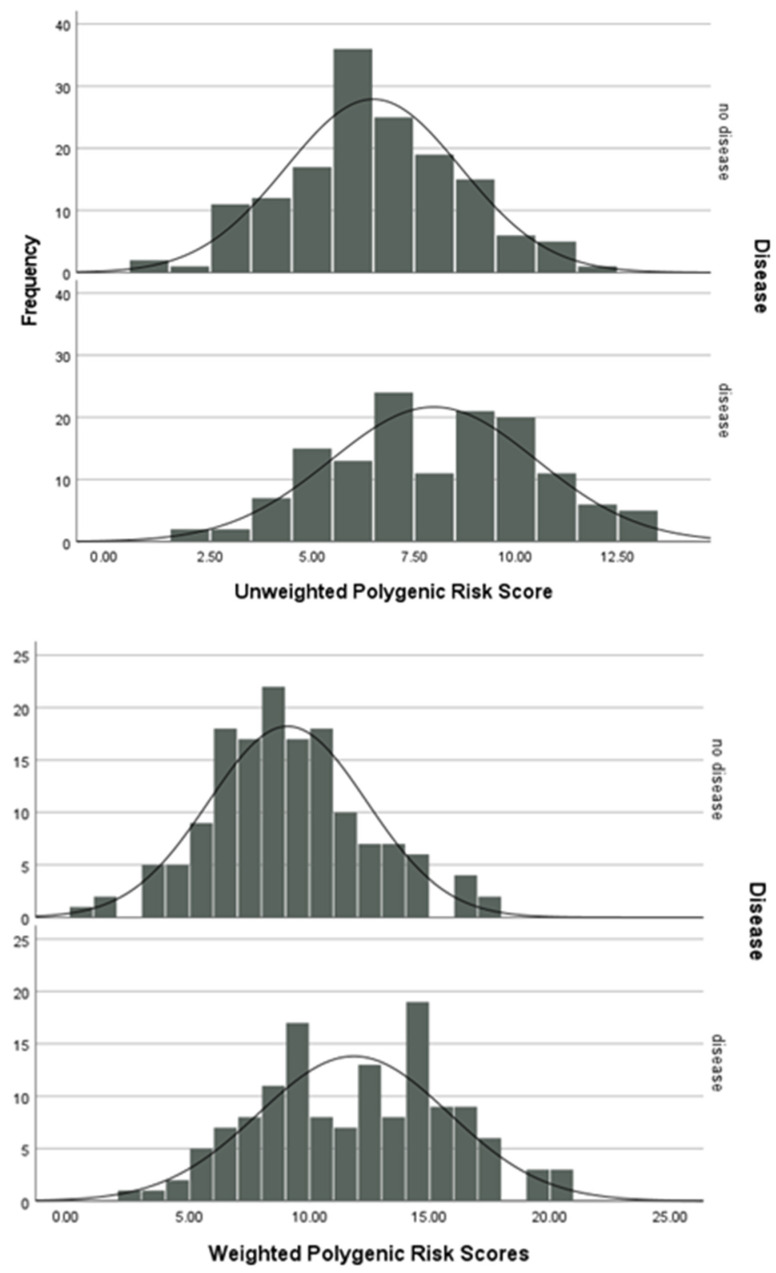
Unweighted and weighted polygenic risk scores.

Polygenic risk scores were used to investigate the cumulative effect of the polymorphisms analysed in this study. The polygenic risk score is higher for those with RA than those without (Figure 2).

The results of an independent t-test indicate that there is a significant difference between the means of the unweighted (mean difference = 1.48, t_285_ = 5.387, *p* < 0.001) and weighted (mean difference = 2.75, t_285_ = 6.437, *p* < 0.001) polygenic risk scores between the patients and controls.

## 4. Discussion

RA is a complex autoimmune disease that has a substantial genetic predisposition. Specific genetic markers and their associated genotypes have been highlighted to show significant associations with RA, and the accumulative effect of these markers can be assessed using polygenic risk scores. This study investigated 11 genetic markers seen to be associated with RA in a European sample of 150 controls and 137 cases and their contribution to disease susceptibility in RA. The majority of loci analysed in this study were in HWE, and the observed allele frequencies in the sample showed good correspondence with the previously published literature and genomic databases.

*ACE*, *VDR* Bsm1, *VDR* Fok1, *VDR* Taq1, and *STAT4* loci show no significant association with RA in the present study. This can potentially be attributed to the small sample size within the study. Significant associations were observed at all other loci.

TNRF1 and TNRF2 are receptors of TNF, but their exact functions in mediating TNF signalling in RA are unclear [34]. A T/G polymorphism in the TNRF2 gene causes an amino acid change from methionine to arginine which may affect receptor function and therefore TNF-a signalling [31]. A significant association was observed between the G allele (OR = 1.87, CI = 1.26–2.77, *p* = 0.001) and RA, which became even stronger after adjustments (OR = 2.28, CI = 1.37–3.80, *p* = 0.001). A meta-analysis on *TNRF2* (T/G) polymorphism in RA reported a significant association between the G/G genotype and RA risk (OR 2.054, CI 1.30–3.97, *p* = 0.002) [31]. In this study, the GG genotype in the dominant model shows a nearly five-fold increase in risk (OR 4.97), but the confidence interval is relatively large due to the small sample size (Table 4).

IL-4 is an interleukin associated with inflammation. The interleukin is seen to play a role in mediating pro-inflammatory cytokines and the upregulation of anti-inflammatory mediators [41]. However, the IL-4 signalling pathway can also induce pro-inflammatory mechanisms. Dong et al. [41] therefore highlight that the role of IL-4 in RA can be complex and can vary substantially. Some studies have reported a protective role of IL-4 in disease susceptibility. The −590 (T/C) promoter polymorphism has been associated with the susceptibility of RA; however, due to the multifactorial nature of the gene, the effect of the polymorphism is not fully understood.

The T/T genotype showed significant susceptibility with RA (OR = 5.17, CI = 1.10–24.36, *p* = 0.05) and the T allele (OR = 1.77, CI = 1.14–2.73, *p* = 0.009). These results are comparable to the meta-analysis results for this locus (OR = 1.30, CI = 1.09–1.55, *p* = 0.003) [32].

TRAF1 encodes proteins that mediate TNF-a signal transduction, which is associated with T-cell proliferation and activation. C5 is a key member of the complement pathway in the immune response, which has been highlighted in association with RA. The G/A polymorphism at hand links to an intergenic region which influences the structure, function, and expression of *TRAF1* or C5 [35]. High ORs for RA with significance across crude and adjusted analyses were seen for *TRAF-C5*. The AA genotype (adjusted OR: 3.53 (95% CI 1.45–8.61) *p* = 0.009) and A allele presence (adjusted OR: 1.78 (95% CI 1.16–2.74) *p* = 0.008) were associated with increased RA risk.

PTPN22 is a negative regulator of T-cell activation. The C/T polymorphism leads to a change from tryptohan to arginine, leading to a reduced interaction between protein and C-terminal Src tyrosine kinase (Csk), increasing unnecessary T- cell activation [35]. PTPN22 and its association with RA has been extensively researched, with a meta-analysis of 125 RA-related SNPs which reported *PTPN22* showing the strongest association for all three models of inheritance [42]. The *PTPN22* (rs2476601) polymorphism showed significant association with RA in both crude and adjusted analyses. The T allele was linked to an over two-fold increase in disease risk (adjusted OR: 2.63 (95% CI 1.45–4.75) *p* = 0.0008 †), and the CT genotype was also associated with increased RA (adjusted OR: 2.21 (95% CI 1.10–4.47) *p* = 0.0024 †); these associations were significant even after Bonferroni correction for multiple comparisons.

HLA associations with RA have been extensively researched since the 1980s, and their association with the disease has been well established, with more than 80% of PA patients carrying at least one of the disease-associated *HLA-DRB1* alleles. The GG genotype was associated with significantly increased RA risk (adjusted OR: 3.67 (CI 95% 1.20–11.19) *p* = 0.02). The presence of the G allele was also linked to increased RA risk in crude and adjusted analyses (adjusted OR: 1.92 (CI 95% 1.20–3.06) *p* = 0.0051). This SNP requires further investigation due to contrasting findings in the literature.

TNFa -308 has been suggested to be a potent signalling molecule, inducing other pro-inflammatory molecules. It is produced by cells present in joints and regulates the production of *IL-1*. TNF receptors have a similar distribution in RA joints, suggesting that TNF can stimulate excessive upregulation of pro-inflammatory responses. The A/A genotype showed a significant association with RA risk (OR = 3.54, CI = 1.25–10.02, *p* = 0.01), as did the A allele (OR = 1.49, CI = 1.02–2.18, *p* = 0.036), but after adjustments for covariates, neither of these associations was significant. Previous research on the *TNF-⍺-*308 SNP observed contrasting results between ethnicities, including the A allele being associated with increased RA in Latin Americans, showing no association in Europeans, and showing the opposite association in Egyptians [43,44].

### 4.1. Polygenic Risk Scores

PRS are a recent method for assessing the cumulative effect of genotypes and their association with disease. As visible in Figure 2, those with RA have a higher genetic risk score than the control group, suggesting their collection of genotypes in the 11 genetic loci puts them at a higher risk of developing RA and therefore shifting the distribution to the right. The results of an independent t-test indicate that there is a significant difference between the means of the unweighted (mean difference = 1.48, t_285_ = 5.387, *p* < 0.001) and weighted (mean difference = 2.75, t_285_ = 6.437, *p* < 0.001) polygenic risk scores between the case and control groups. However, to ensure the avoidance of inequities in health outcomes, wider PRS analysis should be carried out on a large and diverse population [45].

### 4.2. Limitations, Strengths and Clinical Applications

There are some limitations to our study. The sample size was relatively small. Low mutant allele frequencies for several genes also precluded comprehensive analyses, whilst low sample sizes meant many confidence intervals were extensive and insignificant as a result. Thus, as aforementioned, a larger sample size may allow clearer inferences to be drawn. Only a limited number of lifestyle and clinical variables were available for inclusion in genetic covariate adjustments. Additional risk factors such as air pollution, for which exposure to silica or asbestos and living near a road have been linked to RA risk [46], may also be included and adjusted, but these were not available or collected in this study. Exercise levels could also be considered in how these impact RA progression and severity [47]. Furthermore, the potential impacts of epigenetic adaptations and linkage disequilibrium may impact genetic associations and could be considered in further research on SNPs. Epigenetic changes which could impact RA risk, including methylation of genes in the adaptive immune system, have been observed [48], although further understanding of the impact this could pose is required.

Strengths of this study include addressing gaps in the current literature by analysing new polymorphisms for a specific population and adjusting for potential confounders to genetic association results. The study also highlights the cumulative contribution of different genetic loci using a limited PRS score which may have further implications for personalised and clinical medicine.

## 5. Conclusions

This study of European RA patients and controls showed a significant association of seven genetic loci with RA. Six of these associations were reported to significantly increase the risk of RA in the presence of the risk allele (TNRF2*G, TRAF1*A, PTPN22*T, HLA-DRB1*G, *TNF*α*A, and IL4-590*T). Polygenic risk scores for RA patients were significantly higher than for controls. However, the results of this study cannot be utilized for wider clinical applications, and a more representative sample including several ethnicities would be required for clinical and medical usage.

## Figures and Tables

**Table 2:** Participant characteristics for various lifestyle variables.

Variable	Patients N, (%)	Controls N (%)	*p* Value (*t*-Test or Chi-Squared)
Age (average)	49.0	58.9	<0.0001
Sex			
Male	37 (27%)	64 (43%)	0.0047
Female	100 (73%)	84 (57%)	
Diet			
Vegetarian	5 (5%)	15 (11%)	
Mixed	90 (76%)	96 (64%)	0.043
Mostly Meat	22 (19%)	15 (10%)	
Alcohol units per week			
0	79 (57%)	61 (41%)	
1–6	33 (24%)	47 (31%)	<0.0001
7–13	11 (8%)	14 (9%)	
14+	14 (10%)	27 (18%)	

**Table 3:** Allele frequencies, genotype frequencies, and Hardy–Weinberg equilibrium.

Polymorphism	Group	Allele Frequencies		Genotype Frequencies			HWE *p*-Value
*ACE*	Patients	I = 0.44	D = 0.56	I/I = 0.28	I/D = 0.32	D/D = 0.4	0.86
	Controls	I = 0.45	D = 0.55	I/I/ = 0.19	I/D = 0.51	D/D = 0.3	0.01
*VDR* Bsm1	Patients	A = 0.5	T = 0.5	A/A = 0.28	A/T = 0.43	T/T = 0.28	0.15
	Controls	A = 0.54	T = 0.46	A/A = 0.33	A/T = 0.41	T/T = 0.26	0.052
*VDR* Fok1	Patients	T = 0.58	C = 0.42	C/C = 0.18	C/T = 0.48	T/T = 0.33	0.29
	Controls	T = 0.61	C = 0.39	C/C = 0.17	C/T = 0.43	T/T = 0.37	1
*VDR* Taq1	Patients	A = 0.64	G = 0.36	A/A = 0.4	A/G = 0.48	G/G = 0.12	0.096
	Controls	A = 0.66	G = 0.34	A/A = 0.47	A/G = 0.38	G/G = 0.14	0.85
*TNRF2*	Patients	T = 0.59	G = 0.41	G/G = 0.14	T/G = 0.53	T/T = 0.33	0.099
	Controls	T = 0.72	G = 0.28	G/G = 0.05	T/G = 0.46	T/T = 0.49	0.63
*IL4*-591	Patients	C = 0.78	T = 0.22	C/C = 0.63	C/T = 0.31	T/T = 0.07	1
	Controls	C = 0.86	T = 0.14	C/C = 0.74	C/T = 0.25	T/T = 0.01	0.22
*STAT4*	Patients	G = 0.76	T = 0.24	G/G = 0.52	G/T = 0.47	T/T = 0.01	0.006
	Controls	G = 0.73	T = 0.27	G/G = 0.49	G/T = 0.48	T/T = 0.03	0.001
*TRAF1*	Patients	G = 0.46	A = 0.54	A/A = 0.29	G/A = 0.5	G/G = 0.21	0.12
	Controls	G = 0.62	A = 0.38	A/A = 0.11	G/A = 0.54	G/G = 0.35	1
*PTPN22*	Patients	C = 0.77	T = 0.23	C/C = 0.59	C/T = 0.35	T/T = 0.06	1
	Controls	C = 0.88	T = 0.12	C/C = 0.77	C/T = 0.22	T/T = 0.01	0.81
*HLA-DRB1*	Patients	A = 0.64	G = 0.36	A/A = 0.4	A/G = 0.47	G/G = 0.13	0.66
	Controls	A = 0.75	G = 0.25	A/A = 0.55	A/G = 0.4	G/G = 0.05	1
*TNF-α*	Patients	G = 0.7	A = 0.3	A/A = 0.11	G/A = 0.37	G/G = 0.52	0.47
	Controls	G = 0.78	A = 0.22	A/A = 0.03	G/A = 0.37	G/G = 0.6	0.22

**Table 4:** Genetic association analysis using different models of inheritance.

Gene/ SNP	Analysis Model	Genotype/ Allele	Crude	*p*	Adjusted §	*p*
			OR		OR (95% CI)	
*ACE*	Co-dominant	II	1.0 (reference)		1.0 (reference)	
		ID	0.43 (0.23–0.80)	0.006 *	0.60 (0.28–1.30)	0.17
		DD	0.91 (0.48–1.73)		1.11 (0.49–2.50)	
	Log-additive/allelic	D	1.02 (0.75–1.39)	0.90	1.10 (0.74–1.65)	0.63
	Recessive	II-ID	1 (reference)		1 (reference)	
		DD	1.56 (0.94–2.56)	0.08	1.55 (0.82–2.93)	0.17
	Dominant	II	1 (reference)		1 (reference)	
		ID-DD	0.61 (0.94–2.56)	0.08	0.78 (0.38–1.59)	0.49
*VDR*-Bsm1	Co-dominant	AA	1 (reference)		1 (reference)	
		AT	1.24 (0.69–2.24)	0.69	1.78 (0.84–3.81)	0.22
		TT	1.30 (0.68–2.51)		1.05 (0.45–2.43)	
	Log-additive/allelic	T	1.14 (0.82–1.59)	0.42	1.03 (0.68–1.57)	0.88
	Recessive	AA-AT	1 (reference)		1 (reference)	
		TT	1.15 (0.66–2.01)	0.62	0.74 (0.36–1.50)	0.40
	Dominant	AA	1 (reference)		1 (reference)	
		AT-TT	1.26 (0.74–2.17)	0.39	1.46 (0.73–2.91)	0.29
*VDR* Fok1	Co-dominant	TT	1 (reference)		1 (reference)	
		CT	1.34 (0.79–2.26)	0.55	1.13 (0.59–2.17)	0.92
		CC	1.24 (0.63–2.47)		0.99 (0.43–2.28)	
	Log-additive/allelic	C	1.15 (0.82–1.60)	0.41	1.02 (0.68–1.52)	0.93
	Recessive	TT-CT	1 (reference)		1 (reference)	
		CC	1.06 (0.57–1.96)	0.86	0.93 (0.43–2.02)	0.86
	Dominant	TT	1 (reference)		1 (reference)	
		CT-CC	1.31 (0.80–2.14)	0.28	1.08 (0.59–1.97)	0.79
*VDR* Taq1	Co-dominant	AA	1 (reference)		1 (reference)	
		AT	1.47 (0.88–2.45)	0.29	1.46 (0.78–2.14)	0.41
		TT	1.01 (0.48–2.13)		0.85 (0.34–2.10)	
	Log-additive/allelic	T	1.11 (0.79–1.57)	0.53	1.04 (0.68–1.58)	0.86
	Recessive	AA-AT	1 (reference)		1 (reference)	
		TT	0.84 (0.42–1.68)	0.61	0.70 (0.30–1.65)	0.42
	Dominant	AA	1 (reference)		1 (reference)	
		AT-TT	1.34 (0.83–2.17)	0.22	1.28 (0.71–2.30)	0.41
*STAT4*	Co-dominant	GG	1 (reference)		1 (reference)	
		GT	1.36 (0.85–2.17)	0.14	1.59 (0.88–2.85)	0.15
		TT	5.21 (0.57–47.74)		5.33 (0.42–68.07)	
	Log-additive/allelic	T	1.47 (0.95–2.28)	0.08	1.68 (0.97–2.91)	0.06
	Recessive	GG-GT	1 (reference)		1 (reference)	
		TT	4.48 (0.50–40.55)	0.13	4.30 (0.34–54.27)	0.24
	Dominant	GG	1 (reference)		1 (reference)	
		GT-TT	1.42 (0.89–2.25)	0.14	1.65 (0.92–2.94)	0.09
*IL4*-590	Co-dominant	CC	1 (reference)		1 (reference)	
		CT	1.39 (0.82–2.34)		1.49 (0.78–2.87)	0.14
		TT	5.17 (1.10–24.36)	0.047 *	3.95 (0.73–21.44)	
	Log-additive/allelic	T	1.77 (1.14–2.73)	0.0091 *	1.83(1.08–3.10)	0.02 *
	Recessive	CC-CT	1 (reference)		1 (reference)	
		TT	5.17 (1.10–24.35)	0.018 *	3.47 (0.65–18.67)	0.12
	Dominant	CC	1 (reference)		1 (reference)	
		CT-TT	1.73 (1.04–2.87)	0.032 *	1.89 (1.02–3.53)	0.04 *
*TNFR2*	Co-dominant	TT	1 (reference)		1 (reference)	
		TG	1.67 (1.00–2.79)	0.005 *	2.34 (1.21–4.54)	0.004 *†
		GG	4.16 (1.60–10.81)		4.97 (1.44–17.14)	
	Log-additive/allelic	G	1.87 (1.26–2.77)	0.001 *	2.28 (1.37–3.80)	0.001 *
	Recessive	TT-TG	1 (reference)		1 (reference)	
		GG	3.14 (1.26–7.79)	0.009 *	3.27 (1.00–10.68)	0.04 *
	Dominant	TT	1 (reference)		1 (reference)	
		TG-GG	1.91 (1.16–314)	0.009 *	2.64 (1.40–4.99)	0.002 *†
*TRAF1-C5*	Co-dominant	GG	1 (reference)		1 (reference)	
(rs10818488)		AG	0.85 (0.53–1.35)	0.85 (0.53–1.35)	1.19 (1.45–8.61)	1.19 (1.45–8.61)
		AA	3.2 (1.71–5.98)	0 *†	3.53 (1.45–8.61)	0.009 *
	Log-additive/allelic	A	1.99 (1.39–2.84)	0.0001 *†	1.78 (1.16–2.74)	0.008 *
	Recessive	GG-AG	1 (reference)		1 (reference)	
		AA	3.23 (1.73–6.03)	0.0001 *†	3.15 (1.46–6.79)	0.002 *†
	Dominant	GG	1 (reference)		1 (reference)	
		AG-AA	1.98 (1.16–3.36)	0.011 *	1.57 (0.83–2.99)	0.16
*PTPN22*	Co-dominant	CC	1 (reference)		1 (reference)	
		CT	2.06 (1.21–3.50)	0.003 *†	2.21 (1.10–4.47)	0.002 *†
		TT	5.60 (1.16–27.08)	0.003 *†	14.22 (1.56–129.73)	0.002 *†
	Log-additive/allelic	T	2.15 (1.36–3.38)	0.0005 *†	2.63 (1.45–4.75)	0.0008 *†
	Recessive	CC-CT	1 (reference)		1 (reference)	
		TT	4.50 (0.94–21.60)	0.04	10.89 (1.22–97.57)	0.008 *
	Dominant	CC	1 (reference)		1 (reference)	
		CT-TT	2.26 (1.35–3.79)	0.0012 *†	2.68 (1.36–5.29)	0.004 *†
*HLADBR1*	Co-dominant	AA	1 (reference)		1 (reference)	
(rs660895)		AG	1.35 (0.84–2.18)	0.26	1.92 (1.03–3.59)	0.02 *
		GG	2.61 (1.09–6.27)	0.046 *	3.67 (1.20–11.19)	0.02 *
	Log-additive/allelic	G	1.73 (1.19–2.52)	0.004 *†	1.92 (1.20–3.06)	0.005 *
	Recessive	AA-AG	1 (reference)		1 (reference)	
		GG	2.61 (1.09–6.27)	0.026 *	2.69 (0.92–7.84)	0.06
	Dominant	AA	1 (reference)		1 (reference)	
		AG-GG	1.83 (1.14–2.94)	0.012 *	2.15 (1.18–3.90)	0.011 *
*TNF ⍺-*308	Co-dominant	GG	1 (reference)		1 (reference)	
		AG	1.01 (0.63–1.64)	0.95	0.63 (0.33–1.18)	0.09
		AA	3.54 (1.25–10.02)	0.022 *	2.32 (0.64–8.44)	0.09
	Log-additive/allelic	A	1.49 (1.02–2.18)	0.036 *	1.00 (0.62–1.59)	0.99
	Recessive	GG-AG	1 (reference)		1 (reference)	
		AA	3.54 (1.25–10.02)	0.01 *	2.73 (0.77–9.64)	0.10
	Dominant	GG	1 (reference)		1 (reference)	
		AG-AA	1.38 (0.86–2.20)	0.18	0.77 (0.43–1.40)	0.40

* Significant at 5% level before correction. † Significant after Bonferroni correction (*p* < 0.0045). § Adjusted for sex, age, diet, smoking status, and alcohol consumption. Significantly associated with increased RA risk. Significantly associated with increased RA risk, with a wide confidence interval (4+ times OR). Significantly associated with reduced RA risk.

## Data Availability

The study datasets and protocols of the current manuscript are available from the corresponding author upon request.
